# Genome-Wide Association Study for *Haemonchus contortus* Resistance in Morada Nova Sheep

**DOI:** 10.3390/pathogens11080939

**Published:** 2022-08-19

**Authors:** Simone Cristina Méo Niciura, Magda Vieira Benavides, Cintia Hiromi Okino, Adriana Mercia Guaratini Ibelli, Alessandro Pelegrine Minho, Sergio Novita Esteves, Ana Carolina de Souza Chagas

**Affiliations:** 1 Embrapa Pecuária Sudeste, São Carlos 13560-970, SP, Brazil,; 2 Embrapa Pecuária Sul, Bagé 96401-970, RS, Brazil; 3 Embrapa Suínos e Aves, Concórdia 89715-899, SC, Brazil

**Keywords:** gastrointestinal nematode control, genotyping, GWAS, molecular markers, ovine, parasite resistance

## Abstract

Among the gastrointestinal nematodes affecting sheep, *Haemonchus contortus* is the most prevalent and virulent, resulting in health problems and production losses. Therefore, selecting sheep resistant to *H. contortus* is a suitable and sustainable strategy for controlling endoparasites in flocks. Here, 287 lambs of the native Brazilian Morada Nova hair sheep breed were subjected to two consecutive artificial infections with *H. contortus* and assessed for fecal egg count (FEC), packed cell volume (PCV), and live weight (LW). Forty-four animals ranked as having extreme resistance phenotypes were genotyped using the Illumina OvineSNP50v3 chip. A case–control genome-wide association study (GWAS) detected 37 significant (*p* < 0.001) markers in 12 ovine chromosomes in regions harboring quantitative trait loci (QTL) for FEC, *Trichostrongylus* spp. adults and larvae, weight, and fat; and candidate genes for immune responses, mucins, hematological parameters, homeostasis, and growth. Four single-nucleotide polymorphisms (SNP; OAR1_rs427671974, OAR2_rs419988472, OAR5_rs424070217, and OAR17_rs401006318) genotyped by qPCR followed by high-resolution melting (HRM) were associated with FEC and LW. Therefore, molecular markers detected by GWAS for *H. contortus* resistance in Morada Nova sheep may support animal selection programs aimed at controlling gastrointestinal nematode infections in flocks. Furthermore, genotyping of candidate genes using HRM qPCR may provide a rapid and efficient tool for animal identification.

## 1. Introduction

Morada Nova is a hair sheep breed characterized by high heat tolerance and adaptation to tropical climatic conditions [1]. Traditionally bred in the Brazilian Northeast region in small farms under extensive production systems, this native breed is also raised in other conditions and regions of Brazil [2]. Despite their small body size, these animals present interesting traits such as prolificacy, early sexual maturity, maternal ability, lack of reproductive seasonality, and resistance to gastrointestinal nematodes [3]. 

The barber pole worm, *Haemonchus contortus*, is the most prevalent and virulent nematode species that affects small ruminants in tropical regions [4]. *H. contortus* is a blood-sucking parasite of the abomasum, which causes anemia, weight loss, and death [5]. Considering the negative impact of nematodes on animal health and production, and the consequent economic losses [4,6], parasite resistance is an important trait that should be explored for animal selection in sheep. Furthermore, selection for resistance is a sustainable measure for parasite control [7], as it reduces production losses, reduces the use of anthelmintics, and decreases the infectiousness of pastures and the subsequent larval challenge to animals in the flock [8,9].

Several gene polymorphisms associated with resistance to gastrointestinal nematodes have been detected in Morada Nova sheep by candidate gene genotyping [10,11]; however, no genome-wide association studies (GWAS) have been identified for parasite resistance in this breed. Parasite resistance is a quantitative complex trait, and several genes may contribute to the final phenotype [12]. Therefore, GWAS can detect associated genes and polymorphisms, which may help to elucidate the genetic mechanisms affecting the inheritance of parasite resistance. 

Therefore, a GWAS of *H. contortus*-resistant and -susceptible Morada Nova sheep was performed using a case–control design to identify genomic regions involved in resistance to gastrointestinal nematodes. Furthermore, to our knowledge, this was the first GWAS related to *H. contortus* resistance in a Brazilian native parasite-resistant Morada Nova sheep breed resulting in 37 significant markers located on 12 ovine chromosomes harboring regions previously associated with resistance-related traits and functional candidate genes. In addition to providing guidance for studies in other breeds, knowledge of the molecular mechanisms involved in parasite resistance will support the use and maintenance of Morada Nova sheep as a genetic stock for gene introgression and direct use in production systems, aiming to control nematode infections in flocks.

## 2. Results

**Figure 1:**
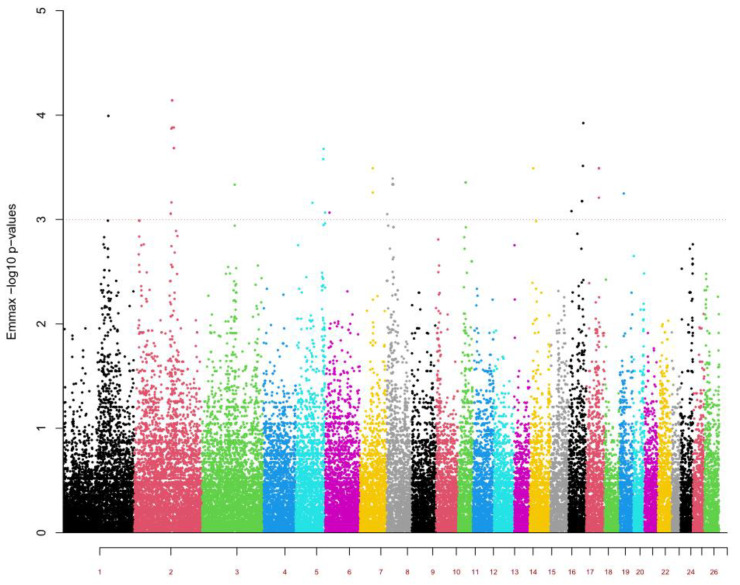
Manhattan plot of genome-wide association study (GWAS) for *Haemonchus contortus* resistance in Morada Nova sheep genotyped using the OvineSNP50v3 chip. Threshold (dashed red line) at Bonferroni *p*-value log_10_ ≥ 3, corresponding to significance at 0.05.

The genome-wide association study (GWAS) identified 37 significant (*p* < 0.001) single nucleotide polymorphism (SNP) markers located on 12 ovine chromosomes (OAR 1, 2, 3, 5, 6, 7, 8, 11, 15, 17, 18, and 20) (Figure 1, Table 1, and Supplementary Table S1).

**Figure 2:**
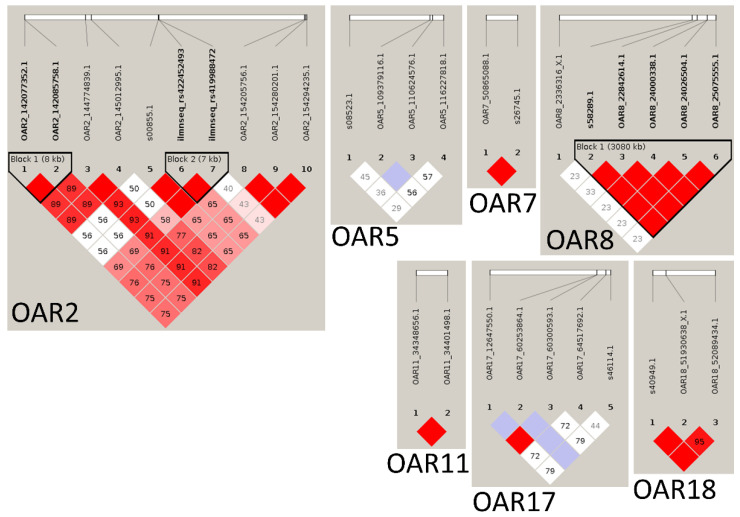
Haploview-generated linkage disequilibrium (LD) plots of markers in GWAS for *H. contortus* resistance in Morada Nova sheep. Three haplotype blocks (bold) were identified for single-nucleotide polymorphism (SNP) markers in OAR 2 (two LD blocks) and OAR 8. Red diamonds without a number indicate complete LD for SNP markers in OAR 7, 11, and 18.

Using Haploview, linkage disequilibrium (LD) blocks were detected for markers located in OAR 2 and 8, and strong LD was observed for markers in OAR 7, 11, and 18, (Figure 2).

In the 2 Mbp upstream and downstream regions of each significant marker, 683 unique genes (Supplementary Table S2), including 431 protein-coding genes, were identified. The same genomic regions harbored reported quantitative trait loci (QTL; Supplementary Table S3) for *Nematodirus*, *H. contortus*, and *Trichostrongylus colubriformis* fecal egg counts (FEC), larvae and adults of *Trichostrongylus* spp. in the abomasum and small intestine, weight, and fat traits (Table 1). 

Functional annotation analyses revealed enrichment of sulfotransferase activity and RNA polymerase II transcription factor activity, sequence-specific DNA binding terms for gene ontology molecular function, visual perception, response to stimulus, and phototransduction terms for biological processes, and homeobox for domain (Supplementary Table S4). Glycosaminoglycan biosynthesis–heparan sulfate/heparin, beta-alanine metabolism, histidine metabolism, and glycolysis/gluconeogenesis were enriched KEGG (Kyoto Encyclopedia of Genes and Genomes) pathways (Supplementary Tables S4 and S5).

The biological roles of genes (with localization on chromosomes) potentially involved in nematode resistance in Morada Nova sheep (Table 1) are related to the following:

- Immune response: *BTLA* (1:175586573–175619634), *CD96* (1:174690709–174790572), *CD200* (1:175479877–175498685), *CFI* (6:15708801–15753015), *DPP4* (2:146632572–146713094), *FER* (5:105044035–105461672), *LEF1* (6:17197574–17304587), *NFE2L2* (2:131754249–131759072), and the *TREML1*, *TREM1*, and *TREM2* gene cluster (20:15158837–15237353);
- Gastric mucosa and mucins: *GALNT10* (5:63016723–63244040), *GCNT3* (7:47666417–47667739), and *PGC* (20:15636993–15649730);
- Hematological parameters: *CLEC14A* (18:47277443–47278737), *DLX1* (2:136599373–136602848), *EDNRA* (17:10416386–10482226), and *SH2B3* (17:54732694–54757389);
- Homeostasis: *CCDC80* (1:175729335–175762809), *HS3ST5* (8:23301492–23469323), and *PLA2G1B* (17:62282828–62288877);
- Growth and muscle development: *DPPA2* (1:172525876–172536184), *HOXD* gene cluster (2:132820016–132915560), *KLHL41* (2:138842626–138856360), *MYF5* (3:116620277–116622399), and *PITX2* (6:14934427–14955991);
- Lipid metabolism and fat deposition: *MAPK7* (11:33629196–33632861) and *SREBF1* (11:34176887–34191779).

**Figure 3:**
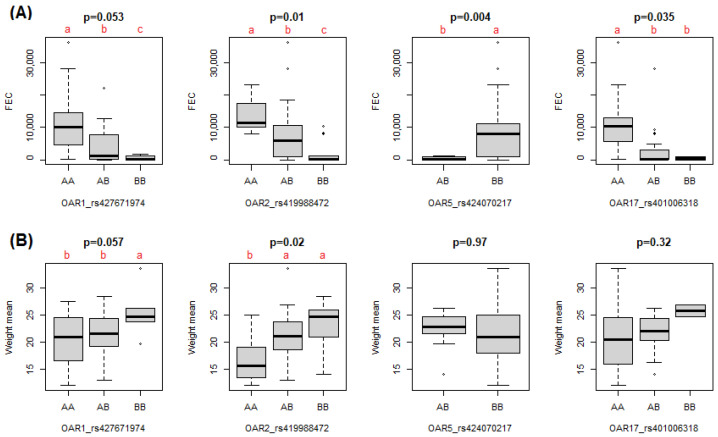
Box plots showing the genotypic effects of SNP markers (rs427671974, rs419988472, rs424070217, and rs401006318 in OAR 1, 2, 5, and 17, respectively) on phenotypic traits in Morada Nova sheep, with *p*-values obtained by ANOVA. Different letters in red represent differences in group means according to Tukey’s test. (**A**) Mean fecal egg count (FEC). (**B**) Mean live weight.

Real-time polymerase chain reaction (qPCR) followed by high-resolution melting (HRM) analyses confirmed the genotypes attributed using the chip in all 44 animals for OAR1_rs427671974, OAR2_rs419988472, OAR5_rs424070217, and OAR17_rs401006318, demonstrating 100% agreement. In addition, these four SNPs were associated with FEC (Figure 3A), and OAR1_rs427671974 and OAR2_rs419988472 were associated with live weight (LW) (Figure 3B). No association with packed cell volume (PCV) was detected for any of the four SNP markers.

## 3. Discussion

The present study employed a case–control GWAS for *H. contortus* resistance using OvineSNP50v3 chip genotyping of 44 ranked phenotyped (considering FEC, PCV, and LW) animals from a population of 287 Morada Nova sheep, with data from two experimental parasite challenges. GWAS detected 37 significant molecular markers, some in strong LD, on 12 ovine chromosomes in genomic regions harboring several functional candidate genes and QTLs for related traits. GWAS was validated by HRM qPCR genotyping of four significant SNP markers that were associated with FEC and LW. Mixed gastrointestinal parasite species are present under natural or field infection [12], and uninfected animals can be confounded by highly resistant animals [13]. Then, the use of two experimental parasite challenges, comprising monospecific artificial infection with *H. contortus* and repetitive phenotype collection, may have contributed to the suitability of the employed GWAS approach.

Significant markers were located in genomic regions harboring QTLs for parasite resistance, such as *H. contortus*, *T. colubriformis*, and *Nematodirus* FEC and *Trichostrongylus* spp. adults and larvae in the abomasum and small intestine, in OAR 2, 3, 7, 8, 11, 17, and 18. In addition, superposition of production trait QTLs, such as weight and fat, was detected in OAR 1, 2, 3, 5, 6, 8, 11, and 18. An association with production traits was expected in the present study, as live weight was used to rank animals with extreme phenotypes. However, several studies based solely on FEC and nematode infection rates have also detected regions spanning production QTLs, suggesting a correlation between live weight and growth with resistance [14], as well as a natural selection effect to ensure developmental stability under the challenge from gastrointestinal nematode infection [13]. Some genomic regions detected in GWAS for *H. contortus* resistance in Morada Nova sheep did not harbor reported QTLs for related traits, as observed for OAR 5, 15, 17, and 20, confirming that different molecular mechanisms may regulate resistance in different breeds. Consequently, due to the genetically fragmented nature of sheep and goat populations, GWAS information derived from one breed cannot be extrapolated to others without proper validation [7]. 

Regarding the enriched terms detected in functional analyses, homeobox has previously been associated with adaptive immune response in cattle [15], and the glycosaminoglycan biosynthesis heparan sulfate/heparin pathway has been associated with viral invasion (reviewed by [16]). In addition, changes in the metabolism of beta-alanine and other amino acids, mainly due to the effect of microbiota on the host, were observed following *H. contortus* infection [17]. 

Several mechanisms can disrupt parasite establishment and confer host resistance to gastrointestinal nematodes [18,19]. Briefly, while feeding in the abomasum, *H. contortus* secretes and excretes antigens that stimulate host inflammatory, humoral, and cellular immune responses. Consequently, the recruited T-helper cells release cytokines, mainly interleukins, which activate IgE synthesis, eosinophils, mast cells, and globular leukocytes in the mucosa. These events are followed by B-cell activation and antibody production (IgA and IgG1). Additionally, mast cell and eosinophil inflammatory products, such as histamines, proteases, leukotrienes, and prostaglandins, lead to mucus production and smooth muscle contraction, which induce parasitic paralysis, elimination, or death. Furthermore, events favoring host homeostasis and coping with parasitic loads, such as protein and energy metabolism, hematological parameters, and body weight, have also been associated with resistance and/or resilience to gastrointestinal nematodes [8,20]. Based on these physiological functions, candidate genes were investigated in the genomic windows 2 Mbp upstream and downstream of each significant SNP marker detected via GWAS. 

Among candidate genes related to the immune response, the expression of *BTLA*, an immunoglobulin superfamily member, and *LEF1*, an enhancer of T-cell receptor-alpha, was increased in peripheral blood mononuclear cells in Suffolk sheep following exposure to *H. contortus* larvae antigens [21]. In addition, *BTLA* expression in host T CD4(+) cells and innate leukocytes affected intestinal immunity and *Strongyloides ratti* infection [22]. Increased expression of *CFI*, which regulates the complement cascade, was detected in the abomasum of *H. contortus*-resistant sheep breeds [23,24,25], and *CFI* was also associated with *H. contortus* FEC [26]. Increased expression of *CD96*, an immunoglobulin superfamily member, has been detected in nematode-susceptible goats [27] and cattle [28]. *CD200* [29], a glycoprotein containing two immunoglobulin domains, and *NFE2L2* (or *Nrf2*) [30], which is involved in the response to oxidative stress, affected macrophage regulation and *Leishmania* infection. *TREML1* (or *TLT-1*), *TREM2*, and *TREM1* genes, which are involved in inflammatory, innate, and adaptive immune responses, were associated with tick resistance in cattle [31], and the role of *TRML1* in clot formation and inflammatory or immune-induced bleeding has been reported [32,33]. Furthermore, increased expression of *TREM1* was detected in sheep peripheral blood mononuclear cells during chronic infection with *Fasciola hepatica* [34]. *FER*, a tyrosine kinase involved in leukocyte recruitment, regulated the intestinal epithelial lipopolysaccharide barrier in response to bacteria [35]. *DPP4*, which is involved in metabolism and immune regulation, was associated with the innate immune response to virus [36] and demonstrated a role in hypoxia response in sheep [37].

Regarding the gastric mucosa and mucins, the *GALNT10* gene, which drives mucin-type O-glycan synthesis, is a paralog of *GALNTL6*, which was found by GWAS to be associated with gastrointestinal parasite resistance in sheep [12]. In addition, decreased expression of *PGC*, a component of the gastric mucosa, has been detected in the abomasum of a sheep breed resistant to *H. contortus* [23]. In cattle, increased expression of *GCNT3*, which plays a role in mucin-type glycoproteins, has been detected in the small intestine in response to *Cooperia oncophora* [38] and in the abomasum following *Ostertagia ostertagi* infection [39]. 

Considering that *H. contortus* is a hematophagous parasite, genes affecting hematological parameters in hosts may have a potential role in resistance. *DLX1*, a homeobox transcription factor, regulated the TGF-β superfamily during blood production [40]; *EDNRA*, which encodes the receptor for endothelin-1, affected vasoconstriction in yaks [41]; *CLEC14A* controlled angiogenesis in mice [42]; and *SH2B3*, a negative regulator of cytokine signaling, was found to be associated with erythrocyte traits in sheep by GWAS [43]. 

Regarding homeostasis, *PLA2G1B*, a phospholipase A2 that regulates energy metabolism and inflammation in the intestine, was considered an endogenous anthelmintic that induces *Heligmosomoides polygyrus* and *Nippostrongylous brasiliensis* death in mice [44]. *HS3ST5*, a cell-surface heparan sulfate, acted as a receptor facilitating *Trypanosoma cruzi* [45] and *Toxoplasma gondii* [46] invasion. In addition, the expression of *CCDC80*, which enables glycosaminoglycan binding activity, was affected by *Trypanosoma cruzi* infection [47]. 

Some of the identified genes in this study were associated with growth, muscle development, and fat traits. Homeobox genes *HOXD1*, *HOXD3*, *HOXD10*, *HOXD12*, and *HOXD13* have been detected by GWAS for muscularity in cattle [48], and *PITX2* was related to growth in sheep [49] and weight in cattle [50]. *DPPA2* was involved in myogenesis [51], *KLHL41* was associated with skeletal muscle differentiation [52], and *MYF5*, a myogenic factor, was associated with growth in sheep [53]. Furthermore, *MAPK7* was involved in adipocyte differentiation [54], and *SREBF1* affected fat metabolism and deposition in sheep [55]. 

The analytical strategy employed by GWAS to detect *H. contortus* resistance in Morada Nova sheep was validated by association analyses of four significant SNP markers genotyped by HRM qPCR with FEC and LW in animals. No association with PCV was detected. However, while FEC and LW were used as factors multiplied by 0.4 to rank animals in extreme phenotypes, PCV presented a lower weight (0.2) in the equation. This decision was based on the fact that PCV presented lower variability among animals compared with FEC and LW, suggesting that Morada Nova sheep are resilient, rather than fully resistant to *H. contortus* [56]. The BB alleles of OAR1_rs427671974 and OAR2_rs419988472 were associated with lower FEC and higher LW, and the AB allele of OAR5_rs424070217 and the BB allele of OAR17_rs401006318 were associated with lower FEC. These markers, in addition to validating the GWAS results, may be used for the selection of resistant Morada Nova sheep using HRM qPCR genotyping.

## 4. Materials and Methods

### 4.1. Parasitological Tests and Phenotypic Classification of Animals

For phenotypic evaluation, 2 g of feces was collected from the rectum, mixed with 28 mL of sodium chloride-saturated solution, and evaluated in a McMaster chamber [57]. The total number of eggs was multiplied by 50 to obtain the fecal egg count (FEC). To determine packed cell volume (PCV), blood was collected into a heparin microcapillary tube and centrifuged at 1200 rpm for 5 min to determine the percentage of erythrocytes. 

The DNA samples and phenotypic data (FEC, PCV, and live weight) used to rank animals with extreme phenotypes were obtained in 2017 and 2018 [58]. Briefly, 287 Morada Nova lambs (146 males and 141 females), the progeny of 7 rams from the Embrapa Pecuária Sudeste flock, were treated with monepantel (2.5 mg/kg; Zolvix^®^) to remove natural infection with gastrointestinal nematodes. After two FEC at 7-day intervals, animals were experimentally infected with 4000 *H. contortus* third-stage larvae (L_3_) from an anthelmintic susceptible isolate [58]. FEC was performed weekly 21, 28, 35, and 42 days post-infection (DPI), PCV was evaluated biweekly on 14, 28, and 42 DPI, and live weight (LW) was obtained on 0, 28, and 42 DPI. At 42 DPI, lambs were dewormed with monepantel (2.5 mg/kg), and, after 15 days, they were subjected to a second parasitic challenge with 4000 L_3_ *H. contortus* of the same isolate, followed by the sampling protocol described previously. At 42 DPI of the second parasitic challenge, mean FEC, PCV, and LW were calculated from the collected data. Subsequently, animals were ranked as extreme phenotypes (extremely resistant and extremely susceptible to *H. contortus*) based on the following equation:

Rank = LW × 0.4 − FEC × 0.4 + PCV × 0.2.(1)

### 4.2. Genome-Wide Association Study (GWAS) 

Based on phenotype ranking, 44 lambs were selected considering homogeneous phenotype distribution (21 resistant and 23 susceptible), sex (23 females and 21 males), and number of progenies (7–12) from four rams with a large number of descendants. This resulted in 12 resistant females, 11 susceptible females, 9 resistant males, and 12 susceptible males. In addition, differences in rank mean value varied from 8 to 36 times between extreme phenotype progenies from each ram. 

DNA was extracted by saline precipitation [59] from venous blood samples obtained from lambs. DNA integrity was confirmed via 1% agarose gel electrophoresis, and the DNA concentration and purity (260/280 absorbance ratio between 1.8 and 2.0) were estimated using a NanoDrop 2000 spectrophotometer. The samples were genotyped using the Illumina OvineSNP50v3 chip (Supplementary Table S6) at the Centro de Genômica Funcional at ESALQ/USP in Piracicaba, SP, Brazil. 

### 4.3. Bioinformatics and Functional Annotation

Genotyping chip data were subjected to quality control filtering, with 47,782 single-nucleotide polymorphisms (SNP) retained for analyses by case–control GWAS [60]. PLINK was used to filter markers, and SNPs were removed if they had a minor allele frequency (MAF) < 0.01, a call rate < 90%, a GC score < 0.6, and a deviation from Hardy–Weinberg equilibrium (HWE) < 10^−^^15^. An efficient mixed-model association expedited (EMMAX) was used to identify nominal *p*-values for phenotype–genotype interactions, and then PLINK (using –assoc, –perm, and –adjust functions) was used for Bonferroni testing. Linkage disequilibrium (LD) between SNP markers was calculated using Haploview [61]. 

Genes located within a 2 Mbp window upstream and downstream of each marker were searched in the OAR v3.1 sheep genome using BioMart (www.ensembl.org/biomart accessed on 28 April 2022) and the Ensembl Genes 106 database. The same genomic interval was investigated for superposition with related sheep quantitative trait loci (QTL) mapped on SheepQTLdb (https://www.animalgenome.org/cgi-bin/QTLdb/OA/index accessed on 13 July 2022) using the Sheep Genome Track on OAR_3.1 (https://www.animalgenome.org/cgi-bin/gbrowse/sheep/ accessed on 13 July 2022). 

ENSEMBL_Gene_ID was used as a gene list in the functional annotation tool of the DAVID Bioinformatics Resources 2021 update (https://david.ncifcrf.gov/home.jsp accessed on 29 April 2022) with *Ovis aries* genome as the background. DAVID was used to identify the Kyoto Encyclopedia of Genes and Genomes (KEGG) pathways [62]. Considering that an enrichment score (ES) of 1.3 is equivalent to *p* = 0.05 in Fisher’s exact test [14], ES > 1.3 was used as the threshold to detect significantly enriched terms. Classification in GeneCards version 5.9 (https://www.genecards.org/ accessed on 9 May 2022) and a literature review were used to identify functional candidate genes. 

### 4.4. GWAS Validation

For GWAS validation, four significant SNP markers (rs427671974, rs419988472, rs424070217, and rs401006318) located on four ovine chromosomes (OAR 1, 2, 5, and 17, respectively), were genotyped via quantitative PCR (qPCR) followed by high-resolution melting (HRM) in the 44 lambs subjected to GWAS. The HRM qPCR assay consisted of 1X SsoFast Evagreen Supermix (Bio-Rad 172–5200), 0.3 µM of each primer (Supplementary Table S7), and 5 ng DNA in a final volume of 10 µL. The thermal profile on a CFX96 thermocycler (Bio-Rad, Hercules, CA, USA) was 95 °C for 3 min, 40 cycles at 95 °C for 10 s and 60 °C for 30 s (reading), followed by melting dissociation curve analysis, from 65 to 95 °C for 10 s with 0.2 °C/5 s increments. HRM analysis was performed using Precision Melt Analysis software (Bio-Rad 184–5015).

The effects of SNPs on FEC, PCV, and LW were analyzed as described by [10]. Briefly, mean FEC values were normalized by orderNorm (ORD) using the bestNormalize package in R. Sex, age at weaning, group, ram, birth type, and age of dam were used as fixed effects, and each SNP was individually tested by ANOVA, using the aov() function, followed by Tukey’s test adjusted for unbalanced group sizes (HSD.test()).

## 5. Conclusions

GWAS to determine *H. contortus* resistance profiles using 44 extreme phenotype Morada Nova sheep genotyped by Illumina OvineSNP50 chip resulted in 37 significant markers in OAR 1, 2, 3, 5, 6, 7, 8, 11, 15, 17, 18, and 20. Marker genomic regions harbored QTLs for FEC, adults and larvae of *Trichostrongylus* spp. in the abomasum and small intestine, weight, and fat traits, in addition to functional candidate genes related to the immune response, gastric mucin, hematological parameters, homeostasis, growth, and fat deposition. HRM qPCR genotyping of four molecular markers (OAR1_rs427671974, OAR2_rs419988472, OAR5_rs424070217, and OAR17_rs401006318) validated their association with FEC and live weight. Thus, the obtained results can be used for the selection of native Morada Nova sheep with the aim of controlling gastrointestinal nematode infection in flocks and increasing parasite resistance in production systems.

## Figures and Tables

**Table 1:** Single-nucleotide polymorphism (SNP) markers associated (*p* < 0.001) with *Haemonchus contortus* resistance in Morada Nova sheep via genome-wide association study (GWAS). Ovine chromosomes (OAR), significance order, EMMAX *p*-value, SNP ID, SNP position (bp), superposition to functional candidate protein-coding genes, and superposition to quantitative trait loci (QTL) in the 2 Mbp upstream and downstream interval.

OAR	Order	*p*-Value	Illumina SNP ID	SNP ID ^1^	Position (bp)	Candidate Protein-Coding Genes ^2^	QTL
1	3	1.02 × 10^−4^	OAR1_187356862.1	**rs427671974**	173891491	*DPPA2*, *CD96*, *CD200*, *BTLA*, *CCDC80*	Weight and fat
2	1	7.22 × 10^−5^	s00855.1	rs409592801	139163515	*KLHL41*	Weight
	2	7.22 × 10^−5^	ilmnseq_rs422452493	rs422452493	139188127		
	5	1.32 × 10^−4^	ilmnseq_rs419988472	**rs419988472**	139195167		
	6	1.32 × 10^−4^	OAR2_154205756.1	rs412327523	145234800	*DPP4*	
	8	2.07 × 10^−4^	OAR2_154280201.1	rs406150872	145308492		
	9	2.07 × 10^−4^	OAR2_154294235.1	rs417376212	145336020		
	7	1.35 × 10^−4^	OAR2_144774839.1	rs424565808	136145355	*NFE2L2*, *HOXD1*, *HOXD3*, *HOXD10*, *HOXD12*, *HOXD13*, *DLX1*	Weight, *Trichostrongylus* spp. adults and larvae in the abomasum, and *Nematodirus* FEC
	30	6.85 × 10^−4^	OAR2_145012995.1	rs430008551	136362050		
	35	8.79 × 10^−4^	OAR2_142077352.1	rs401620358	133594324		
	36	8.79 × 10^−4^	OAR2_142085758.1	rs412805133	133602325		
3	24	4.63 × 10^−4^	OAR3_124041988.1	rs403393991	116331730	*MYF5*	Fat and *T. colubriformis* FEC
5	10	2.11 × 10^−4^	OAR5_110624576.1	**rs424070217**	101668310	-	Weight
	11	2.64 × 10^−4^	OAR5_109379116.1	rs413371484	100428672		
	31	6.92 × 10^−4^	s08523.1	rs411511506	63148146	*GALNT10*	
	33	8.59 × 10^−4^	OAR5_116227818.1	rs398223820	106805224	*FER*	-
6	34	8.59 × 10^−4^	OAR6_19652340.1	rs421701377	16732227	*PITX2*, *CFI*, *LEF1*	Weight and fat
7	15	3.26 × 10^−4^	s26745.1	rs401054470	46306835	*GCNT3*	*H. contortus* FEC
	25	5.52 × 10^−4^	OAR7_50865088.1	rs413854960	46138713		
8	17	4.05 × 10^−4^	OAR8_22842614.1	rs419418467	20206310	*HS3ST5*	Fat and *Trichostrongylus* spp. adults and larvae in the small intestine and abomasum
	20	4.60 × 10^−4^	s58289.1	rs416090516	19586041		
	21	4.60 × 10^−4^	OAR8_24000338.1	rs410048009	21552710		
	22	4.60 × 10^−4^	OAR8_24026504.1	rs421189130	21578631		
	23	4.60 × 10^−4^	OAR8_25075555.1	rs418914462	22667003		
	37	8.87 × 10^−4^	OAR8_2336316_X.1	rs402371066	2115027	-	
11	18	4.41 × 10^−4^	OAR11_34348656.1	rs410744616	32138419	*MAPK7*, *SREBF1*	Weight, fat, and *Trichostrongylus* spp. adults and larvae in the small intestine
	19	4.41 × 10^−4^	OAR11_34401498.1	rs404901308	32191958		
15	13	3.22 × 10^−4^	OAR15_15781330_X.1	rs412682230	15625639	-	-
17	4	1.19 × 10^−4^	s46114.1	**rs401006318**	60852961	*PLA2G1B*	-
	12	3.06 × 10^−4^	OAR17_64517692.1	rs425080766	59101448		
	28	6.67 × 10^−4^	OAR17_60253864.1	rs399621490	55225820	*SH2B3*	
	29	6.67 × 10^−4^	OAR17_60300593.1	rs410780866	55270864		
	32	8.31 × 10^−4^	OAR17_12647550.1	rs422538638	11361784	*EDNRA*	FEC
18	14	3.23 × 10^−4^	OAR18_51930638_X.1	rs416293834	48707173	*CLEC14A*	Weight and *H. contortus* FEC
	16	3.23 × 10^−4^	OAR18_52089434.1	rs428856771	48867859		
	27	6.17 × 10^−4^	s40949.1	rs403982333	48663303		
20	26	5.63 × 10^−4^	s01331.1	rs406291711	14815776	*TREML1*, *TREM2*, *TREM1*, *PGC*	-

^1^ In bold, SNP ID of four markers detected by GWAS and validated by qPCR followed by high-resolution melting (HRM) genotyping. ^2^ Functional candidates from protein-coding genes located in the 2 Mbp upstream and downstream interval.

## Data Availability

Additional data, not presented in Supplementary Materials, are available on request from the corresponding author.

## Supplementary Materials

The following supporting information can be downloaded at: https://www.mdpi.com/article/10.3390/pathogens11080939/s1, Table S1: EMMAX *p*-values for phenotype–genotype interactions, Table S2: Genes located in the 2 Mbp upstream and downstream regions of each significant SNP marker on ovine chromosomes (OAR), Table S3: Quantitative trait loci (QTL) in the 2 Mbp upstream and downstream regions of each significant SNP marker on ovine chromosomes (OAR), Table S4: Enriched terms in functional annotation analyses using DAVID, Table S5: Enriched KEGG pathways, Table S6: OvineSNP50v3 Illumina, and Table S7: Fragment sizes and primers used in HRM qPCR for genotyping of SNP markers in four ovine chromosomes (OAR).
